# Quantitative Comparison of Effects of Dofetilide, Sotalol, Quinidine, and Verapamil between Human *Ex vivo* Trabeculae and *In silico* Ventricular Models Incorporating Inter-Individual Action Potential Variability

**DOI:** 10.3389/fphys.2017.00597

**Published:** 2017-08-18

**Authors:** Oliver J. Britton, Najah Abi-Gerges, Guy Page, Andre Ghetti, Paul E. Miller, Blanca Rodriguez

**Affiliations:** ^1^Department of Computer Science, University of Oxford Oxford, United Kingdom; ^2^AnaBios Corporation San Diego, CA, United States

**Keywords:** safety pharmacology, dofetilide, sotalol, quinidine, verapamil, cardiac modeling

## Abstract

**Background:** *In silico* modeling could soon become a mainstream method of pro-arrhythmic risk assessment in drug development. However, a lack of human-specific data and appropriate modeling techniques has previously prevented quantitative comparison of drug effects between *in silico* models and recordings from human cardiac preparations. Here, we directly compare changes in repolarization biomarkers caused by dofetilide, dl-sotalol, quinidine, and verapamil, between *in silico* populations of human ventricular cell models and *ex vivo* human ventricular trabeculae.

**Methods and Results:** *Ex vivo* recordings from human ventricular trabeculae in control conditions were used to develop populations of *in silico* human ventricular cell models that integrated intra- and inter-individual variability in action potential (AP) biomarker values. Models were based on the O'Hara-Rudy ventricular cardiomyocyte model, but integrated experimental AP variability through variation in underlying ionic conductances. Changes to AP duration, triangulation and early after-depolarization occurrence from application of the four drugs at multiple concentrations and pacing frequencies were compared between simulations and experiments. To assess the impact of variability in IC50 measurements, and the effects of including state-dependent drug binding dynamics, each drug simulation was repeated with two different IC50 datasets, and with both the original O'Hara-Rudy hERG model and a recently published state-dependent model of hERG and hERG block. For the selective hERG blockers dofetilide and sotalol, simulation predictions of AP prolongation and repolarization abnormality occurrence showed overall good agreement with experiments. However, for multichannel blockers quinidine and verapamil, simulations were not in agreement with experiments across all IC50 datasets and I_Kr_ block models tested. Quinidine simulations resulted in overprolonged APs and high incidence of repolarization abnormalities, which were not observed in experiments. Verapamil simulations showed substantial AP prolongation while experiments showed mild AP shortening.

**Conclusions:** Results for dofetilide and sotalol show good agreement between experiments and simulations for selective compounds, however lack of agreement from simulations of quinidine and verapamil suggest further work is needed to understand the more complex electrophysiological effects of these multichannel blocking drugs.

## Introduction

Cardiotoxicity is a major cause of attrition during drug development (Piccini et al., 2009). The current difficulty of predicting cardiotoxic effects of new drug candidates plays a major role in the termination of drug development programmes (Cook et al., 2014). Currently, the pro-arrhythmic potential of a candidate drug is assessed preclinically using a combination of an *in vitro* hERG channel assay and *in vivo* animal cardiovascular studies (Anon, 2005a), followed by a Thorough QT study—an ECG-based study of cardiac repolarization in the later stages of drug development (Anon, 2005b; Wiśniowska et al., 2017). While this strategy has been effective in preventing approval and marketing of new drugs with strongly pro-arrhythmic potential (Ewart et al., 2014; Vargas et al., 2015), QT prolongation alone is an imperfect marker for fatal pro-arrhythmic effects (Shah, 2005) and can result in ending development of safe drugs (Stockbridge et al., 2013; Polak et al., 2015).

The Comprehensive *in vitro* Pro-Arrhythmia Assay (CiPA), a public-private collaboration with the aim of updating the existing cardiac safety testing paradigm, has been proposed to improve the assessment of new drug candidates' pro-arrhythmic risk (Sager et al., 2014). CiPA will consist of multiple components including an ion channel screen of seven channels, combined with an *in silico* modeling component that will model the effect of new drugs on a human ventricular action potential (AP) using data from the ion channel screens (Colatsky et al., 2016; Fermini et al., 2016). Therefore, *in silico* modeling is likely to soon become part of mainstream pro-arrhythmic risk assessment in drug development (Rodriguez et al., 2016; Li et al., 2017; Windley et al., 2017).

Recently, several modeling methodologies have been developed to address simulating the effects of different sources of variability and the effect this has on the response of cardiomyocytes to drugs. In particular, methodologies have been developed to integrate the large amount of inter-individual variability present in electrophysiological recording, which is hypothesized to contribute to inter-individual variability of drug response, with traditional cardiac modeling that uses a single model representative of average cardiomyocyte behavior (Sarkar and Sobie, 2010; Davies et al., 2012; Britton et al., 2013; Sadrieh et al., 2013; Groenendaal et al., 2015). Methods are also under development to probabilistically quantify the high levels of uncertainty in measured drug IC50 values (Mirams et al., 2014; Johnstone et al., 2016).

However, the lack of human-specific data and appropriate modeling techniques have prevented assessment of the degree to which *in silico* models can predict potentially pro-arrhythmic drug-induced changes to the cardiac AP, including change to quantitative biomarkers such as action potential duration (APD) and triangulation (Hondeghem et al., 2001), and the occurrence of qualitative phenomena such as early after-depolarizations (EADs) (Qu et al., 2013). The ability to predict these cellular biomarkers of pro-arrhythmic risk underpins the use of *in silico* modeling to predict pro-arrhythmic risk for new drugs.

In this study, we systematically and quantitatively compare drug-induced changes in repolarization biomarkers predicted by human ventricular cell models against changes observed from AP recordings of human ventricular trabeculae (Page et al., 2016). We investigate four drugs commonly used as reference drugs, three of which are torsadogenic: dofetilide; dl-sotalol; and quinidine, and verapamil, which is a non-torsadogenic drug. Both the average drug response and the variability in drug response are compared against experiments using populations of models (Britton et al., 2013, 2017; Muszkiewicz et al., 2016) to mimic observed inter- and intra-heart variability in AP biomarkers through variability in underlying ion channel densities. The O'Hara-Rudy (ORd) ventricular cell model (O'Hara et al., 2011), which has been selected by a consensus of *in silico* modelers for use in CiPA's *in silico* assay (Colatsky et al., 2016; Fermini et al., 2016), is used as the baseline model for the populations of models. Multiple recent datasets measuring drug block using both standard IC50-based approaches (Kramer et al., 2013; Crumb et al., 2016) and state- and voltage-dependent models of hERG block (Li et al., 2017) are used to obtain simulation results from a variety of drug block models. We identify areas of qualitative and quantitative agreement and disagreement between simulations and this specific set of experiments and discuss strategies for interpreting the results of *in silico* drug response predictions.

We find that quinidine and verapamil produce substantial disagreement between experiments and simulations across multiple concentrations, IC50 datasets, and hERG block models, while dofetilide and sotalol have generally good agreement between experiments and simulations in both degree of AP prolongation and development of repolarization abnormalities.

## Methods

### Experimental data acquisition

Microelectrode AP recordings from stimulated *ex vivo* human ventricular trabeculae at 1 and 2 Hz were obtained as described in detail in Page et al. (2016). Briefly, undiseased donor hearts were obtained from organ donors in the United States with legal consent. Trabeculae were dissected from the inner endocardial wall of the ventricle and used for microelectrode recording at ~37°C. Each trabecula was paced under control conditions to establish a baseline for that trabecula for each frequency, and then three increasing concentrations of drug were applied. For each step of this protocol, trabeculae were paced at both 1 and 2 Hz. For this study, we used the baseline control recordings and recordings from the two higher concentrations of each drug, as at the lowest concentration of each drug the effect of the drug was small compared to experimental variability. In addition, only data from left ventricular trabeculae were used, to remove electrophysiological differences between left and right ventricles as a source of variability. All donor hearts used in this study included recordings from at least three left ventricular trabeculae. Examples of AP traces used in this study are shown in Figure S1 in the Supplementary Material.

### Baseline human ventricular cell model

The ORd model (O'Hara et al., 2011) of the human ventricular cardiomyocyte was used as the baseline model for our investigations, as it is particularly well-suited for studying human ventricular repolarization; is one of the most recent, widely used and extensively tested models of the human ventricular cardiomyocyte using experimental recordings from over 140 human hearts; and has been identified as the model to be used in the *in silico* component of CiPA (Colatsky et al., 2016; Fermini et al., 2016).

Models were paced at 1 and 2 Hz using a biphasic stimulus protocol to approximate the electrotonic effects of tissue coupling (Livshitz and Rudy, 2009). Model code is available in the Supplementary Material and includes the modification proposed by Passini et al. (2016) of the I_NaF_ inactivation gate to improve upstroke robustness over different conductance profiles.

### Simulating experimental variability in AP biomarkers through variability in ionic conductances using populations of models

Based on the ORd model and using the methodology described in Britton et al. (2013) and Britton et al. (2017), we first created an initial pool of 20,000 candidate models by varying 11 ionic conductances for the following currents: I_NaF_, I_NaL_, I_CaL_, I_to_, I_Kr_, I_Ks_, I_K1_, I_NCX_, I_NaK_, I_RyR_, and I_SERCA_, with resulting differences in baseline electrophysiological properties and responses to drug application. Each conductance was randomly selected using Latin Hypercube Sampling (McKay et al., 1979) across a range of 0.25–1.75 times the baseline value of that conductance in the original ORd model. This range was selected for two reasons. Firstly, this range allows substantial conductance variability while disallowing extremely low conductance values, which would only be expected to occur under pathological conditions. This reflects the undiseased nature of the human hearts used in this study. Secondly, the range allows up to sevenfold variation in conductances, in line with the range of conductance variability reported from studies of neurons (Schulz et al., 2006). This is an approximation as equivalent measurements for cardiomyocytes have not yet been reported, although variability in the conductances of individual currents in cardiomyocytes are known to be highly variable (Qi et al., 2008; Xiao et al., 2008) and affected by a wide range of external factors including circadian rhythms, hormones and pacing rate (Qi et al., 2008; Jeyaraj et al., 2012; Odening and Koren, 2014). Finally, conductances were independently sampled as no evidence of covariation has been reported. Should advances in experimental methods allow for a better characterization of ionic conductances in intact tissue, these assumptions can be reviewed.

As different hearts were used in different experiments of drug block, we created populations of models based on the AP biomarker ranges for each individual heart. Due to the limited number of trabeculae available for each heart, biomarker ranges were calculated as the minimum and maximum values of each biomarker observed across all trabeculae from that heart at a particular pacing frequency. Ranges were calculated for both 1 and 2 Hz pacing in control conditions. Five AP biomarkers were used for filtering: APD10 (APD at 10% of repolarization); APD30; APD90; triangulation (APD90–APD30); and the maximum negative (repolarization) gradient of membrane potential with respect to time. These biomarkers were selected to focus on accurate representation of the variability during repolarization, without using a large number of biomarkers. The biomarker ranges for some hearts were narrow, and using larger numbers of biomarkers resulted in fewer models being found that were within range for all biomarkers. There was therefore a trade-off between the number of models in each of the final populations and the number of biomarkers that each model in a population was guaranteed to be within the experimental range for.

For each heart, the biomarker ranges calculated from trabeculae from that heart were used to select from the pool of 20,000 candidate models only those models which had all biomarkers within the ranges calculated for that heart, for both pacing frequencies. These models formed a population of models for the heart, where all models in the population had different conductance parameters, representing different possible ionic profiles that all produced AP biomarkers that were consistent with the observed variability between preparations from that heart. The populations of models therefore allow evaluation of predictions of the variability of response to drug application, not just the average response, and allow consideration of a wide range of ionic scenarios. The information content from action potential measurements such as those typically recorded in human-based studies is insufficient to identify the specific conductances of a cardiomyocyte. We therefore chose to analyse a wide range of ionic scenarios that are consistent with experimental recordings. This allows testing the hypothesis that variability in ionic conductances is critical for the comparison of experiments and simulations of drug block.

### Biomarker calculation

Biomarkers were calculated from the final pacing cycle of each simulation, and from the mean of a sequence of 30 pacing cycles from each experimental recording. In simulations, EADs were classified as depolarizations that occurred more than 100 ms after the beginning of a pacing cycle with a voltage gradient >0.01 mV/ms. For recordings, EADs were classified as any abnormal depolarizations during phases 2 or 3 of an AP, after the upstroke completed but before normal repolarization was complete.

### Simulations

Simulations were performed using the CVODE adaptive timestep ODE solver (Hindmarsh et al., 2005) implemented within the CHASTE software package (Pitt-Francis et al., 2008). Data analysis was carried out using Python scripts.

### Simulation of drug effects–simple pore block model

Drug effects were first simulated using a simple pore block model using IC50 and Hill coefficient data. The blocked fraction of a current *I* was calculated as:

$$\begin{array}{l} B=\frac{1}{1+{\left(\frac{C}{IC50}\right)}^{h}}, \end{array}$$

where *B* is the fraction of *I* that is blocked by a compound, *C* is the concentration of the compound, and *IC50* and *h* are the measured IC50 and Hill coefficient of the compound against that current, respectively. *B* was calculated for each simulation, and the conductance of each affected current was multiplied by the unblocked fraction (1 − *B*) to simulate block.

IC50 values have substantial uncertainty attached to them, and there is considerable variability between studies reporting IC50s of the same compounds. We chose to use and compare IC50 values from two recent studies, by Crumb et al. (2016), which assessed six ion channels (hERG—I_Kr_, KvLQT1/mink—I_Ks_, Kv4.3–I_to_, Kir2.1–I_K1_, Nav1.5—I_NaF_ and I_NaL_, and Cav1.2—I_CaL_), and by Kramer et al. (2013), which assessed 3 (hERG—I_Kr_, Nav1.5—I_NaF_, and Cav1.2—I_CaL_). We simulated two separate datasets to indicate whether variability in IC50 values and number of channels assessed substantially altered simulation results.

For each simulation of drug block, only models from the populations that corresponded to hearts that had been used for experiments with that drug were simulated. For each drug, simulations were performed at 1 and 2 Hz, for two different concentrations an order of magnitude apart. The fractional blocks of ionic currents calculated for each drug, concentration, and dataset are listed in Table S1 in the Supplementary Material.

### Simulation of drug effects–dynamic hERG block model

To capture possible changes to effective hERG block caused by the binding kinetics of the drugs used in this study, we also performed repeats of each drug simulation with the ORd model's formulation of I_Kr_ replaced with the state-based model of I_Kr_ and I_Kr_ block developed by Li et al. (2017). Unlike the simple-pore block model, this model of hERG block integrates data on drug-specific binding timescales and degrees of trapping, as well as the steady-state concentration dependence of channel block. Briefly, this model uses a state-transition modeling approach with six unbound states (two closed; two closed and inactivated; one open; and one open and inactivated) and three drug-bound states (open and bound; closed and bound; and inactivated, open and bound). Therefore, transient binding and unbinding of drugs during the AP can be simulated, and a trapping parameter determines the degree to which each drug can prevent a bound open channel from closing. Each drug simulation was repeated using this state-based hERG and hERG block model by replacing the ORd model's I_Kr_ formulation and simple pore block model of I_Kr_ block. In simulations for drugs with multichannel block, the previous drug blocks calculated from the Crumb and Kramer datasets were used for non-I_Kr_ currents. Changes to model biomarkers in control conditions caused by replacing the I_Kr_ model are summarized in Table S2 in the Supplementary Material.

### Statistics

Intra-individual, inter-individual and total variability in biomarker values was assessed using coefficients of variation (CV). Effects on AP biomarkers of drug application were assessed using change relative to control conditions. Drug response data from experiments and simulations are visualized using boxplots. The central box indicates the central quartiles and median of the data. Boxplot whiskers extend to the farthest data point less than two times the interquartile range from the median.

## Results

### Inter-heart APD biomarker variability is of similar magnitude to intra-heart variability

**Figure 1:**
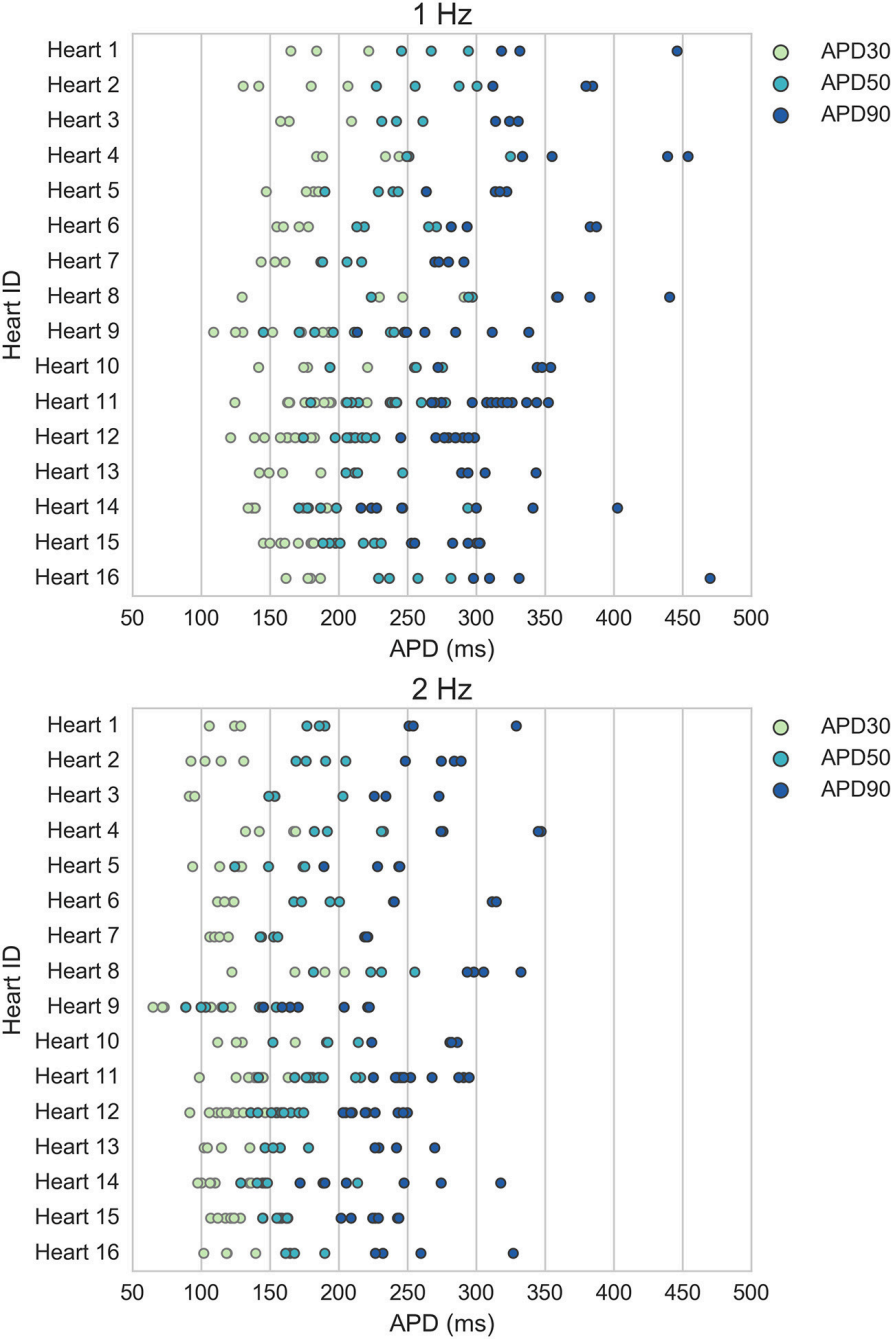
Variability of APD30/50/90 values under control conditions for ventricular trabeculae from different donor hearts. Each dot indicates mean results of 30 action potentials from one trabeculae, each row represents data from a different donor heart. **Top**: 1 Hz pacing. **Bottom**: 2 Hz pacing.

Figure 1 displays the mean APD30, APD50, and APD90 values recorded for each trabecula from the baseline control period of each experiment, for 1 and 2 Hz pacing, grouped by donor heart. Variability between trabeculae from the same heart (intra-individual variability) and between the means of different hearts (inter-individual variability) is quantified in Table 1. CVs for intra- and inter-individual variability were of similar magnitude–neither source of variability made a dominant contribution to total biomarker variability.

**Table 1:** Total, intra- and inter-heart variability.

**Biomarker**	**Mean (*n* = 89)**	**Intra-individual CV**	**Inter-individual CV**	**Total CV**
APD90	312 ms	0.12	0.12	0.17
APD30	171 ms	0.14	0.12	0.18
Triangulation 90–30	141 ms	0.21	0.17	0.28

*Intra-individual variability was defined as the mean of CVs that were each calculated using biomarker values from an individual heart. Inter-individual variability was defined as the CV calculated from the mean biomarker values from each heart. Total variability was defined as the CV calculated from biomarker values from all trabeculae*.

### Development of the populations of models in control conditions

**Figure 2:**
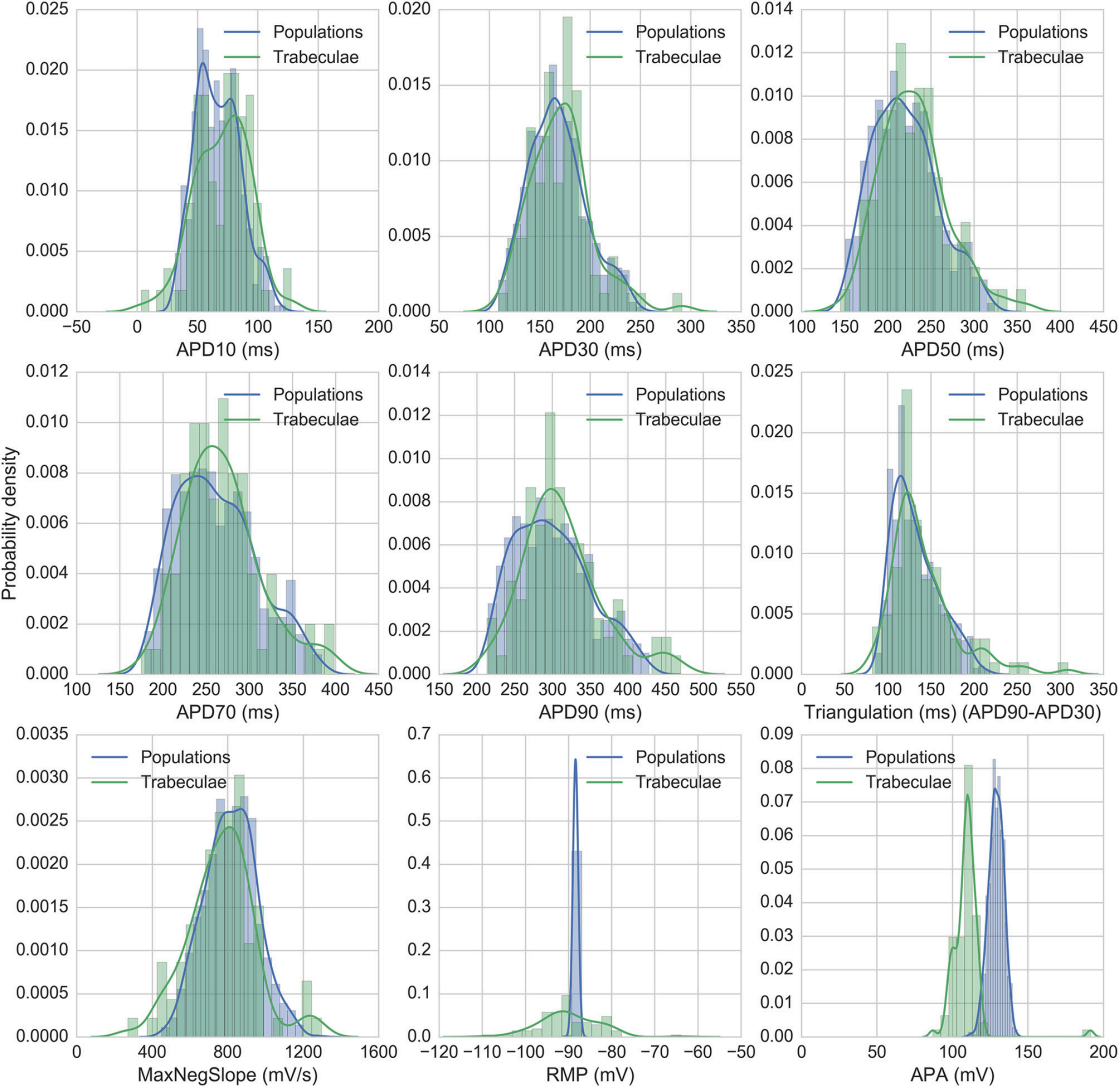
Distributions of experimental and model biomarkers. Normalized histograms and probability density estimates for biomarkers from all trabeculae (green, *n* = 89) and from all populations of models (blue, *n* = 860) under control conditions 1 Hz pacing.

Figure 2 shows the biomarker distributions for the full experimental dataset and the models accepted for nine standard AP biomarkers, including the five biomarkers used for calibration. 860 models were accepted in total across all populations. Model biomarkers show good overlap with the range and shape of the experimental biomarker distribution for seven of the biomarkers, with the two exceptions being resting membrane potential (RMP) and action potential amplitude (APA). RMP is more variable between experiments than between models, which may be due to experimental fluctuations, particularly in extracellular K^+^, that are not modeled in this study. APA (the difference between RMP and peak membrane potential) has similar variability between models and experiments, but the distribution mean is shifted ~+20 mV in the model distribution relative to experiments.

**Figure 3:**
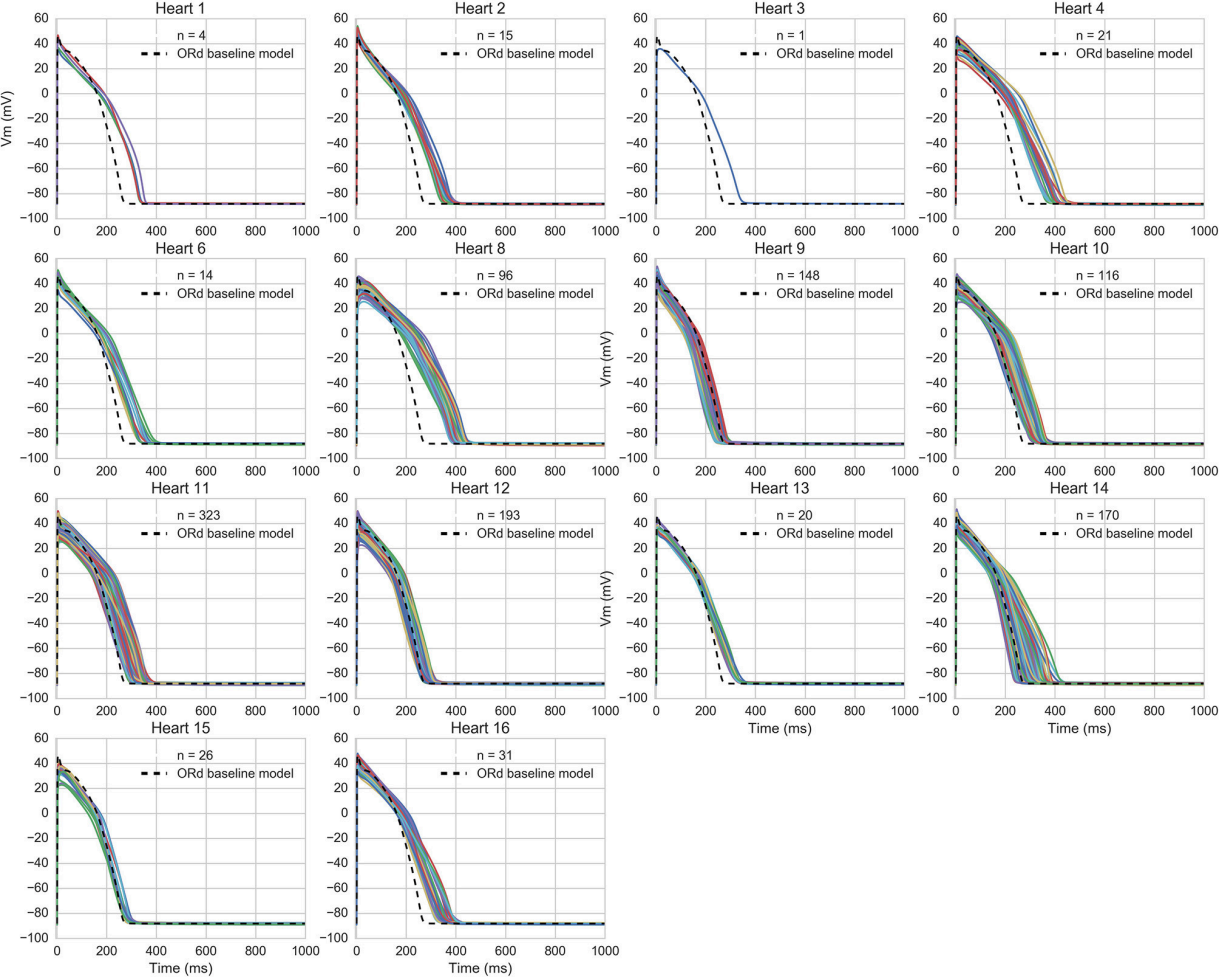
AP traces of models in each heart-specific population of models. AP traces for each heart-specific population of models, and trace from the ORd baseline model for reference. 2/16 hearts did not have any of the 20,000 candidate models in range for all biomarkers and therefore had no accepted models, so are excluded from the figure. 860 out of the 20,000 candidate models were accepted into at least one population.

The accepted models were in range with experimental biomarkers at both frequencies for 14/16 hearts (model APs for each population are shown in Figure 3). The majority of hearts had substantial variability between trabeculae (Figure 1) but for two hearts, the biomarker ranges between experiments were very narrow and none of the 20,000 tested models were within range, simultaneously, for the five tested biomarkers at both 1 and 2 Hz pacing frequencies. The 860 accepted models provided acceptable coverage of the biomarker space for the purpose of our study, which was to allow comparison of drug response between experiments and simulations (rather than to construct a population for every heart).

**Figure 4:**
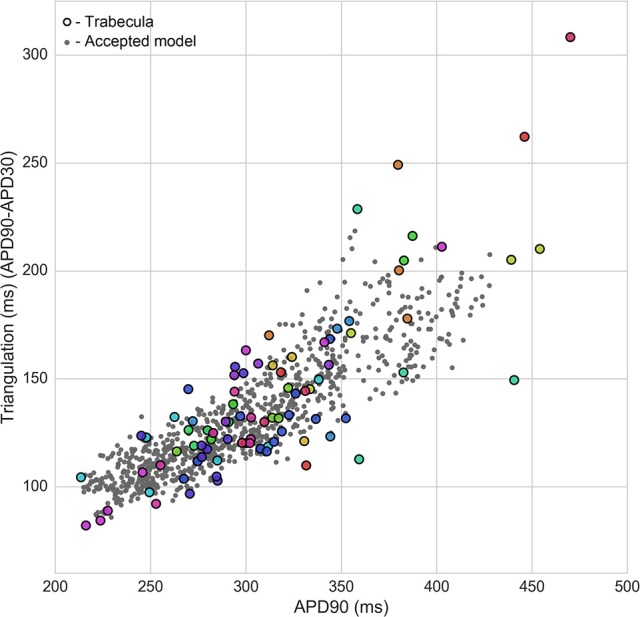
Calibration of heart-specific populations of models using biomarker ranges. Values from all individual trabeculae (colored dots–each color corresponds to a donor heart), and for all models accepted into any population (gray dots) are shown for APD90 vs. triangulation, two of the five biomarkers used to construct the populations, which are also biomarkers of drug-induced pro-arrhythmic risk.

Figure 4 shows the overlap between experimental and model biomarkers for models accepted into all of the populations for APD90 and triangulation, two biomarkers of pro-arrhythmic risk that were also used to calibrate the populations. There is generally good coverage of the experimentally-observed range of biomarkers, potentially highlighting the ability of the ORd model and variability in ionic conductances to account for variability in human electrophysiological measurements, although for the most outlying combinations of biomarkers there were no candidate models that were in range for all five biomarkers at both pacing frequencies simultaneously. This highlights the fact that in spite of the large conductance variability imposed, simulations did not yield the most outlying combinations of biomarker values observed in experiments. Therefore, this type of quantitative comparison also allows identification of potential limitations of the ORd model in capturing outlying behaviors from experiments through variability in conductances, which may require sources of variability beyond ion channel densities to account for the variability in experimental recordings.

### Comparison of experimental and simulated drug application for dofetilide, sotalol, quinidine, and verapamil using multiple Ic50 datasets and I_kr_ models

Using data from studies by Kramer et al. (2013) and Crumb et al. (2016), we simulated application of four reference drugs, three that have high risk torsadogenic classifications (quinidine, dofetilide, dl-sotalol) and one that is classified as low risk, but has a significant hERG IC50 (verapamil).

Quinidine and verapamil, in addition to both being multichannel blocking compounds, are also known to have “untrapped” hERG binding dynamics, which means when bound to hERG they block the channel from closing and so can unbind at polarized membrane potentials (Zhang et al., 1999; Tsujimae et al., 2004; Windley et al., 2017). Depending on the timescales of channel binding and unbinding, this can result in reduced effective block. Due to the potential effects of these binding dynamics, which are not incorporated in the simple pore block model of drug action, we hypothesized that inclusion of these binding dynamics would improve agreement of quinidine and verapamil simulations with experiments, and that not accounting for these effects could result in overestimating the effects of hERG block, as demonstrated in a simulation study by Di Veroli et al. (2014). Therefore, we additionally evaluated the effects of replacing the ORd model's original I_Kr_ model with the recently developed state-based dynamic I_Kr_ model from Li et al. (2017), which includes state- and voltage-dependent drug binding and includes parameterized models for the four drugs used in this study.

**Figure 5:**
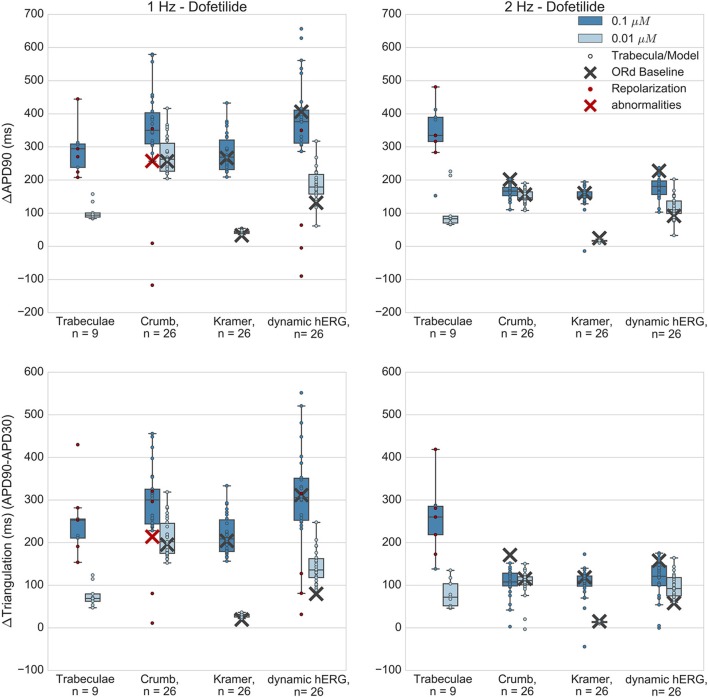
Dofetilide. Changes to APD90 and triangulation relative to control from application of 0.01 and 0.1 μM dofetilide during 1 and 2 Hz pacing. In each panel, response is shown for (left to right): human ventricular trabeculae, populations of models using drug effects calculated using data from Crumb et al. from Kramer et al. and from use of the hERG model by Li et al. As Crumb and Kramer datasets both measured only hERG IC50s for dofetilide, unlike the other tested compounds, there is only one result from use of the dynamic model. Dots indicate results from individual trabeculae and models, crosses show the result from the baseline ORd model. Red symbols indicate simulations and experiments where repolarization abnormalities occurred.

**Figure 6:**
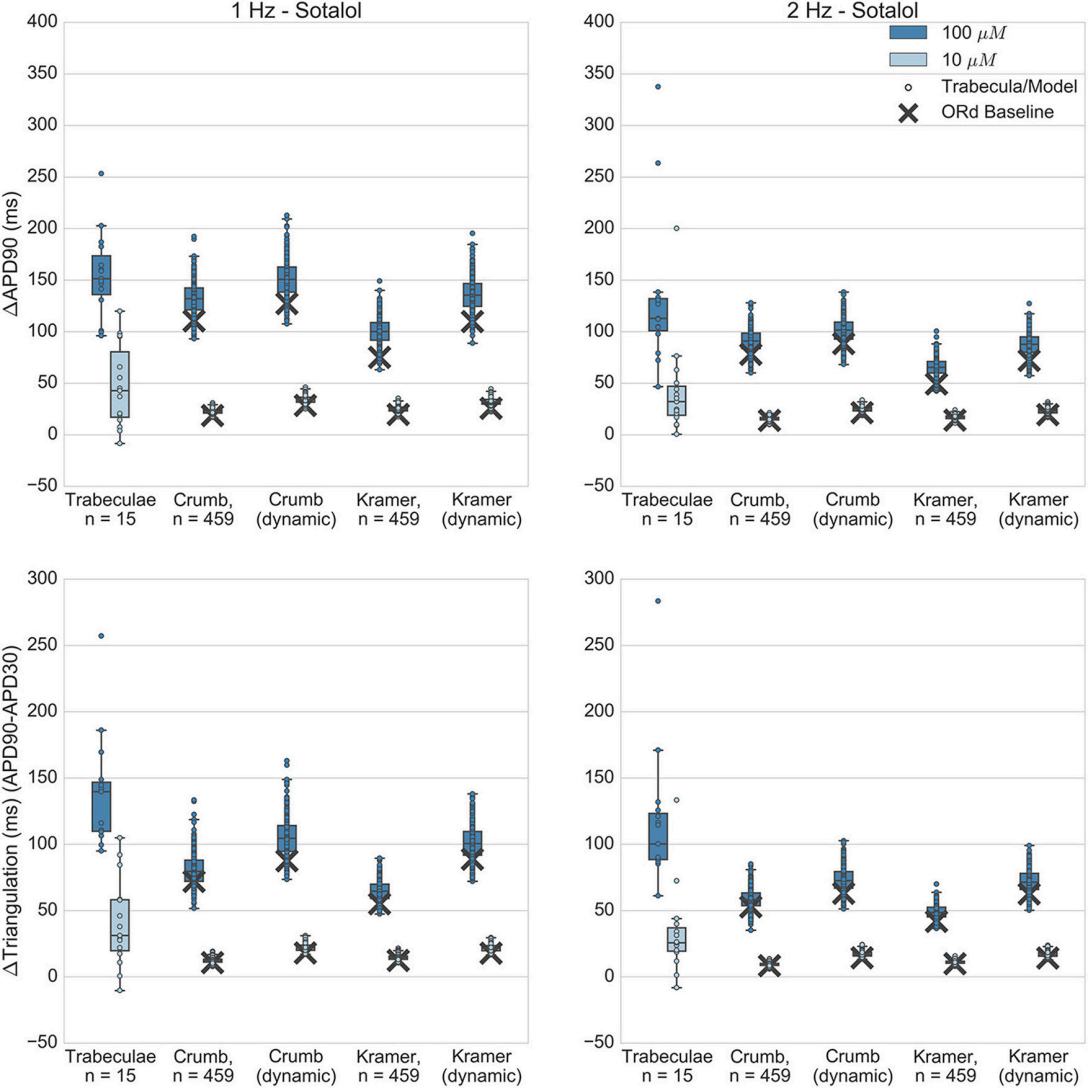
Sotalol. Changes to APD90 and triangulation relative to control from application of 10 and 100 μM sotalol during 1 and 2 Hz pacing. In each panel, response is shown for (left to right): human ventricular trabeculae, populations of models using drug effects calculated using data from Crumb et al. from Crumb et al. with I_Kr_ replaced by the Li et al. I_Kr_ model; from Kramer et al. and from Kramer et al. with I_Kr_ replaced by the Li et al. I_Kr_ model. Dots indicate results from individual trabeculae and models, crosses show the result from the baseline ORd model.

**Figure 7:**
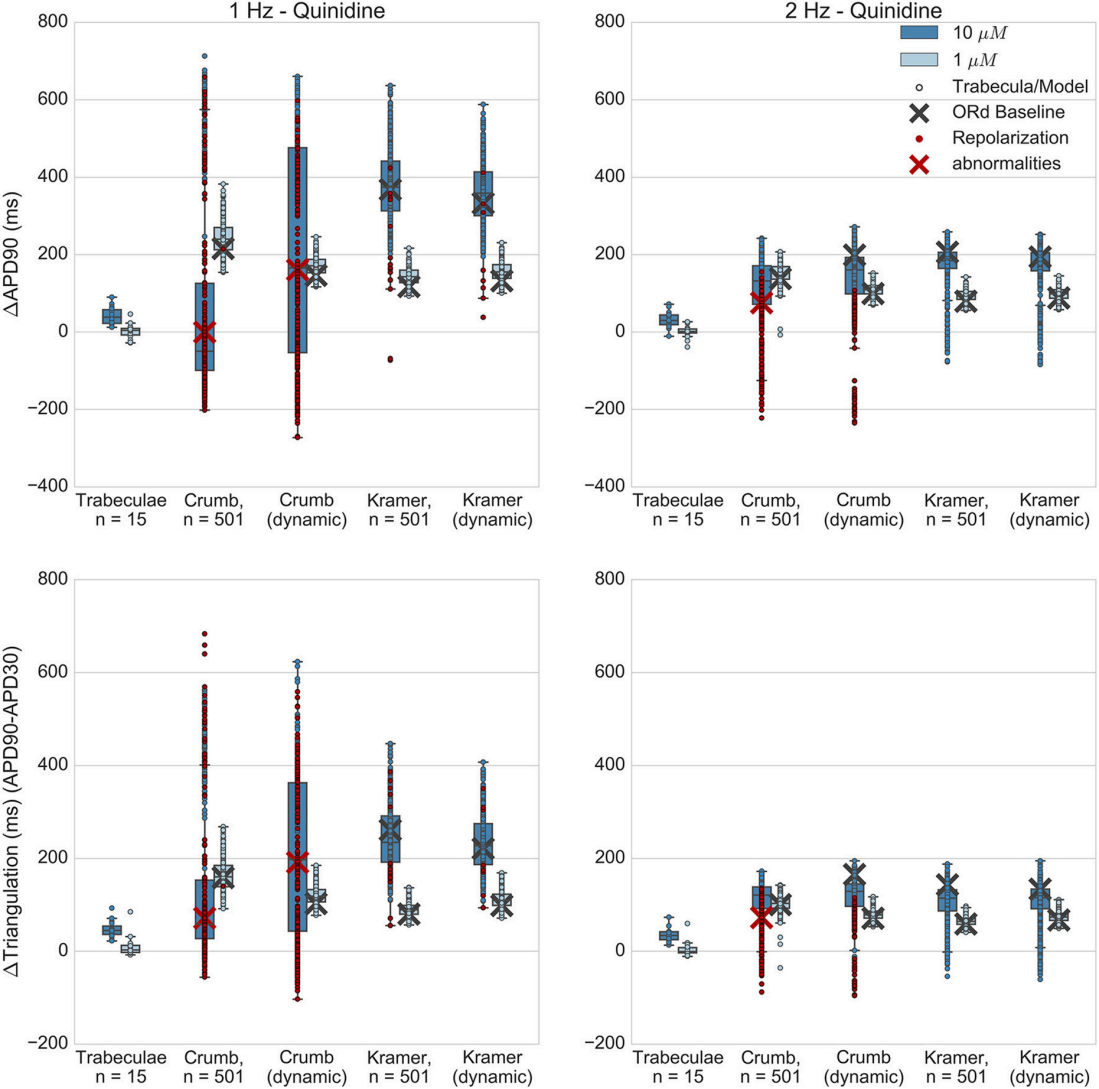
Quinidine. Changes to APD90 and triangulation relative to control from application of 1 and 10 μM quinidine during 1 and 2 Hz pacing. In each panel, response is shown for (left to right): human ventricular trabeculae, populations of models using drug effects calculated using data from Crumb et al. from Crumb et al. with I_Kr_ replaced by the Li et al. I_Kr_ model; from Kramer et al.; and from Kramer et al. with I_Kr_ replaced by the Li et al. I_Kr_ model. Dots indicate results from individual trabeculae and models, crosses show the result from the baseline ORd model. Red symbols indicate simulations and experiments where repolarization abnormalities occurred.

**Figure 8:**
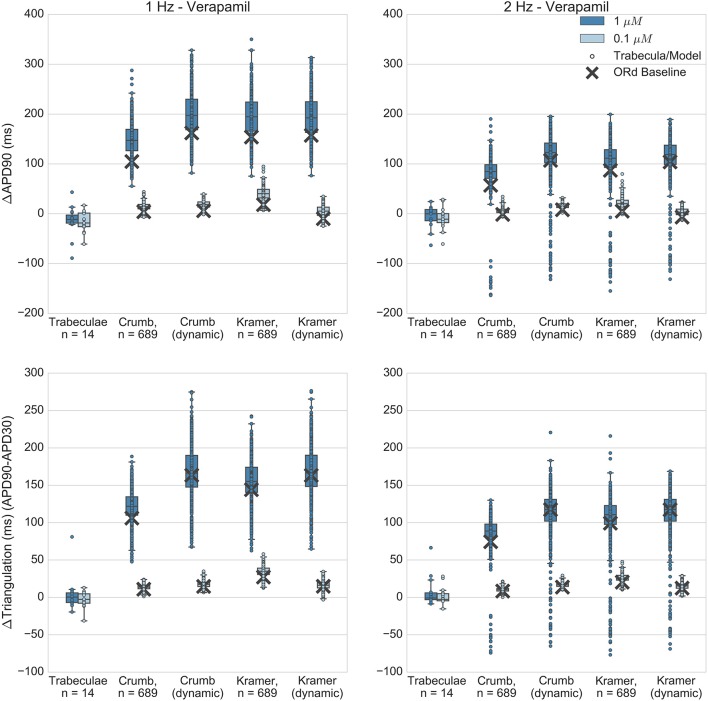
Verapamil. Changes to APD90 and triangulation relative to control from application of 0.1 and 1 μM verapamil during 1 and 2 Hz pacing. In each panel, response is shown for (left to right): human ventricular trabeculae, populations of models using drug effects calculated using data from Crumb et al.; from Crumb et al. with I_Kr_ replaced by the Li et al. I_Kr_ model; from Kramer et al. and from Kramer et al. with I_Kr_ replaced by the Li et al. I_Kr_ model. Dots indicate results from individual trabeculae and models, crosses show the result from the baseline ORd model.

Figures 5–**8** show the changes to repolarization biomarkers APD90 and triangulation under application of each drug for all models in the relevant populations of models for that drug (the populations that were calibrated using data from the hearts that were used in experiments with that drug), and for the original baseline ORd model, compared to experimental results recorded from trabeculae. Figures also indicate models and trabeculae that developed EADs and other repolarization abnormalities (e.g., repolarization failure) under drug application. Biomarker and repolarization abnormality data is additionally summarized in the Supplementary Material (Tables S3–S5).

As expected, there were substantial differences between drugs in the levels of qualitative and quantitative agreement between experiments and simulations. We therefore break down the agreement in changes to APD90, triangulation, and occurrence of EADs between experiments and populations of models using each of the IC50 datasets and I_Kr_ models, for each individual drugs used in this study.

### Dofetilide

Dofetilide is a potent and selective I_*Kr*_ blocker that prolongs the QT interval and is classified as a high-risk compound for drug-induced torsade de pointes (TdP). Qualitatively, application to human trabeculae caused substantial concentration-dependent APD90 and triangulation increase (Figure 5) at both concentrations tested (0.01 and 0.1 μM, Free Therapeutic Concentration (FTC) = 0.002 μM), which was captured by the populations of models and the baseline ORd model using all datasets. EADs occurred in trabeculae from 2/3 tested hearts at 0.1 μM, but did not occur at 0.01 μM. Simulations with the Crumb dataset and the dynamic hERG model both reproduced this behavior at 1 Hz (4/26 models developed repolarization abnormalities in both sets of simulations at 0.1 μM, 0/26 models at 0.01 μM), but no repolarization abnormalities were detected at either concentration using the Kramer dataset, or in any simulation at 2 Hz pacing. The baseline ORd model only developed EADs at 0.1 μM using the Crumb dataset.

Quantitatively, for 0.1 μM dofetilide, APD90 and triangulation changes (ΔAPD and ΔTriangulation) from all datasets were consistent with experiments at 1 Hz pacing, with the distributions of experiments and models overlapping, but not for 2 Hz, where experimental AP prolongation was >1 Hz, unlike all other drugs and concentration studied. In this case, experiments showed prolongation beyond the cycle length. This skipping behavior was not reproduced in any simulations, as the stimulus current was always sufficient to initiate a new AP, while in experiments the stimulus could cause a transient depolarization. Therefore, it is possible that at 2 Hz AP prolongation was >1 Hz due to the additional inward current provided during repolarization by the stimulus.

At the lower dofetilide concentration (0.01 μM), the two IC50 datasets gave substantially different results to each other, neither of which overlapped with the experimental range at 1 Hz. Simulations using the Crumb dataset over-predicted the experimental results, with much higher ΔAPD and ΔTriangulation, and level of variability, than that observed experimentally. In contrast, use of the Kramer dataset under-predicted APD and triangulation increases and variability. However, the dynamic hERG model produced ΔAPD and ΔTriangulation distributions between these two datasets, which did overlap with the experimental range at both pacing frequencies.

Overall, simulations captured the effects of dofetilide—substantial AP prolongation along with incidence of repolarization abnormalities at the higher tested concentration. The comparison with experiments was reasonable for all three sets of simulations at 1 Hz, although no simulations captured the skipping behavior observed at 2 Hz. This could possibly be due to mismatch between experimental and simulated stimulus current strengths. Use of the dynamic hERG model produced the best overall agreement with experiments, as it had overlap with experimental ΔAPD90 and ΔTriangulation ranges for three out of four concentration and frequency combinations (excepting 0.1 μM at 2 Hz), and showed occurrence of repolarization abnormalities.

### Sotalol

Like dofetilide, dl-sotalol is a selective I_Kr_ blocker, although it also has beta-adrenergic receptor blocking effects *in vivo*. Sotalol is torsadogenic and prolongs the QT interval. In the Kramer dataset it was also measured as causing non-negligible block of Cav 1.2 (I_CaL_); however this was not replicated in the Crumb dataset. Application of sotalol caused concentration-dependent APD and triangulation increase in experiments at both tested concentrations (10 and 100 μM, FTC = 14.7 μM), which was captured by all simulations (Figure 6). EADs did not occur at either concentration in any experiments, and this was also reflected in all simulations, as no model developed repolarization abnormalities.

At 100 μM, all sets of simulations displayed overlap with experiments for APD90 increase, although simulations with the Kramer dataset under-predicted the amount of triangulation and APD increase. Overall, results using the dynamic I_Kr_ model were similar to those using the default I_Kr_ model, but for both IC50 datasets use of the dynamic model caused a small increase in APD and triangulation which improved agreement with experiments. For 10 μM, all simulation datasets were fully within the experimental range for both biomarkers, however this was partly because experimental results for 10 μM sotalol displayed much higher variability than simulations. In contrast, for dofetilide, variability at the lower concentration—0.01 μM—was of similar magnitude for experiment and simulations, and less than the variability of the higher concentration—0.1 μM. One potential reason for this is the relatively low I_Kr_ block predicted for 10 μM Sotalol (12.6% from Crumb et al. 14.7% from Kramer et al.) results in a low dispersion of APD prolongation, lower than the intrinsic experimental variability that would be present without drug application, which then dominates the total experimental variability but is not present in simulations.

Overall, sotalol simulations have relatively good agreement with experiments, due to the absence of repolarization abnormalities in all experiments and simulations, and the overlap between simulation and experimental ranges for APD90 and triangulation. The main disagreement between experiments and simulations is that the wide variability observed in both biomarkers at 10 μM in the experiments is uniformly not replicated across all simulations.

### Quinidine

Quinidine is a multichannel blocker, with significant IC50s found for all 3 channels measured in Kramer et al. (Nav1.5/I_NaF_, hERG/I_Kr_, Cav1.2/I_CaL_) and 3/7 of the channels measured by Crumb et al. (hERG/I_Kr_, KvLQT1/I_Ks_, and Kv4.3/I_to_). Crumb et al. also detected block for Nav 1.5 and Cav 1.2 however an IC50 was not reached for these channels during experiments and so was not calculated.

Experimentally, quinidine caused a moderate increase in APD90 and triangulation (Figure 7) at the higher applied concentration (10 μM, FTC = 3.2 μM), and no substantial change at the lower concentration (1 μM) for both 1 and 2 Hz pacing. In addition, no EADs were observed in any experiments. However, 10 μM quinidine had the highest predicted level of hERG block out of all drugs and concentrations tested in this study for both sets of IC50s; the Crumb and Kramer hERG IC50s predicted 95% and 97% I_Kr_ block respectively for 10 μM quinidine (Table S1). In the simulations of quinidine's effects, the Kramer IC50s for quinidine (which measured IC50s for I_NaF_, I_CaL_ and I_Kr_) resulted in higher APD and triangulation increases at both concentrations than were observed experimentally, and EADs were observed in 15/501 models at 10 μM (and none at 1 μM). For the Crumb dataset, in which IC50s were found for I_Ks_, I_to_, and I_Kr_, EADs and other repolarization abnormalities (e.g., repolarization failure) occurred in a large majority of models (421/501) at 10 μM, and for 1/501 models at 1 μM at 1 Hz pacing. The ORd baseline model also developed complete repolarization failure using the Crumb IC50s for 10 μM quinidine. Results for 2 Hz pacing for both sets of IC50s were similar except that no model using the Kramer IC50s developed repolarization abnormalities (Table S5). For 10 μM quinidine, the ΔAPD90 and ΔTriangulation values using the Crumb dataset were highly variable; however these values, particularly from models showing APD shortening, were due to abnormal APs with repolarization abnormalities, rather than due to shortening of normal APs. At 1 μM, the Crumb dataset, like the Kramer dataset, generated much higher levels of APD90 and triangulation increase than seen experimentally.

Quinidine both binds and unbinds rapidly from the hERG channel (Tsujimae et al., 2004; Li et al., 2017; Windley et al., 2017). Therefore, depending on the balance between the timescales of these two processes, there was the possibility that modeling state-dependent block of quinidine would reduce effective AP prolongation. However, results using the dynamic I_Kr_ model with the other measured IC50s produced similar levels of AP prolongation and EAD prevalence compared to the default ORd I_Kr_ model.

Overall, simulations of quinidine predicted far greater APD and triangulation increase (for both Crumb and Kramer datasets and both I_Kr_ block models) than seen in these experiments, and both datasets predicted occurrence of repolarization abnormalities that were also not observed in any trabeculae. The Kramer dataset, which included IC50s for both Nav 1.5 and Cav 1.2 as inward currents, and only hERG as an outward current, still predicted far higher AP prolongation than experiments. Quinidine appears to be a particularly challenging drug to model, which could be due to the wide range of both inward and outward ionic currents that it blocks, and our study identifies that additional experiments are required for its detailed characterization.

### Verapamil

Verapamil blocks both hERG and Cav 1.2 (I_CaL_). Despite blocking hERG, it is non-torsadogenic and is known to have only a small effect on APD and on the QT interval (Johannesen et al., 2014; Vicente et al., 2015) which has been hypothesized to be due to its hERG binding kinetics (Zhang et al., 1999; Di Veroli et al., 2014) and/or counteracting effects of I_CaL_ block.

Recordings obtained with verapamil applied at 0.1 and 1 μM (FTC = 0.081 μM) showed minor APD shortening of similar magnitude at both concentrations (Figure 8), while in all sets of simulations most models produced concentration-dependent APD and triangulation increase, in qualitative disagreement with experiments. A minority of models developed AP shortening, predominantly in 2 Hz simulations. For simulations at the lower concentration (0.1 μM) this was due to drug-induced shortening of normal APs, in line with experimental results. However for simulations at the higher concentration (1 μM) shortening was caused by AP prolongation beyond the duration of the pacing cycle. For these models, the APD was longer than the pacing cycle and so repolarization was incomplete during the next stimulus. This lead to a reduced upstroke and shortened APD on the subsequent pacing cycle. This behavior was not observed in experiments. However, simulations and experiments were in agreement for repolarization abnormality occurrence: no repolarization abnormalities were detected in any experiments or simulations.

Quantitatively, both Crumb and Kramer datasets generated similar distributions of APD and triangulation increase to each other, suggesting that uncertainty in IC50 values is less likely to be the source of the mismatch with experiments for verapamil. Instead, the simple pore drug model and IC50 data used in this study may not be sufficient to approximate the electrophysiological effects of verapamil due to its binding kinetics, and/or the balance of L-type calcium and hERG currents in the ORd model may not be accurate.

Verapamil can unbind from hERG channels at voltages close to typical cardiac resting membrane potentials (Zhang et al., 1999; Windley et al., 2017), although the timescale is relatively slow (time constant of recovery ~100 s at −80 mV). This type of “untrapped” behavior has been shown in simulation studies (Di Veroli et al., 2014) to potentially reduce AP prolongation due to hERG block relative to a “trapped” hERG blocker (e.g., dofetilide). Therefore, this was an important drug to simulate with the dynamic hERG model, as neglect of its unbinding dynamics could potentially cause a substantial overestimation of AP prolongation.

However, Figure 8 shows that use of the dynamic hERG model did not substantially alter predictions of APD prolongation compared to the simple-pore block model using only IC50 data. For example, for the Crumb dataset, mean ΔAPD90 at 1 μM, 1 Hz pacing was 148 ± 32 ms for populations using the ORd I_Kr_ model, 198 ± 43 ms with the dynamic I_Kr_ model, while for the Kramer dataset in the same conditions, with the ORd I_*Kr*_ model ΔAPD90 was 195 ± 40 ms, and 194 ± 43 ms with the dynamic I_Kr_ model. Therefore, across all models simulated, use of a drug block model of I_Kr_ that included data on binding rates and trapping behavior did not improve the agreement of simulated APD prolongation with experimental results (experimental ΔAPD90 in this case was −15 ± 30 ms).

Overall, no set of simulations was consistent with the minor AP shortening caused by verapamil in experiments, as all sets of simulations instead showed AP prolongation. However, all simulations were consistent with the observed lack of repolarization abnormalities.

### Comparison of drug block datasets and modeling methodologies

**Figure 9:**
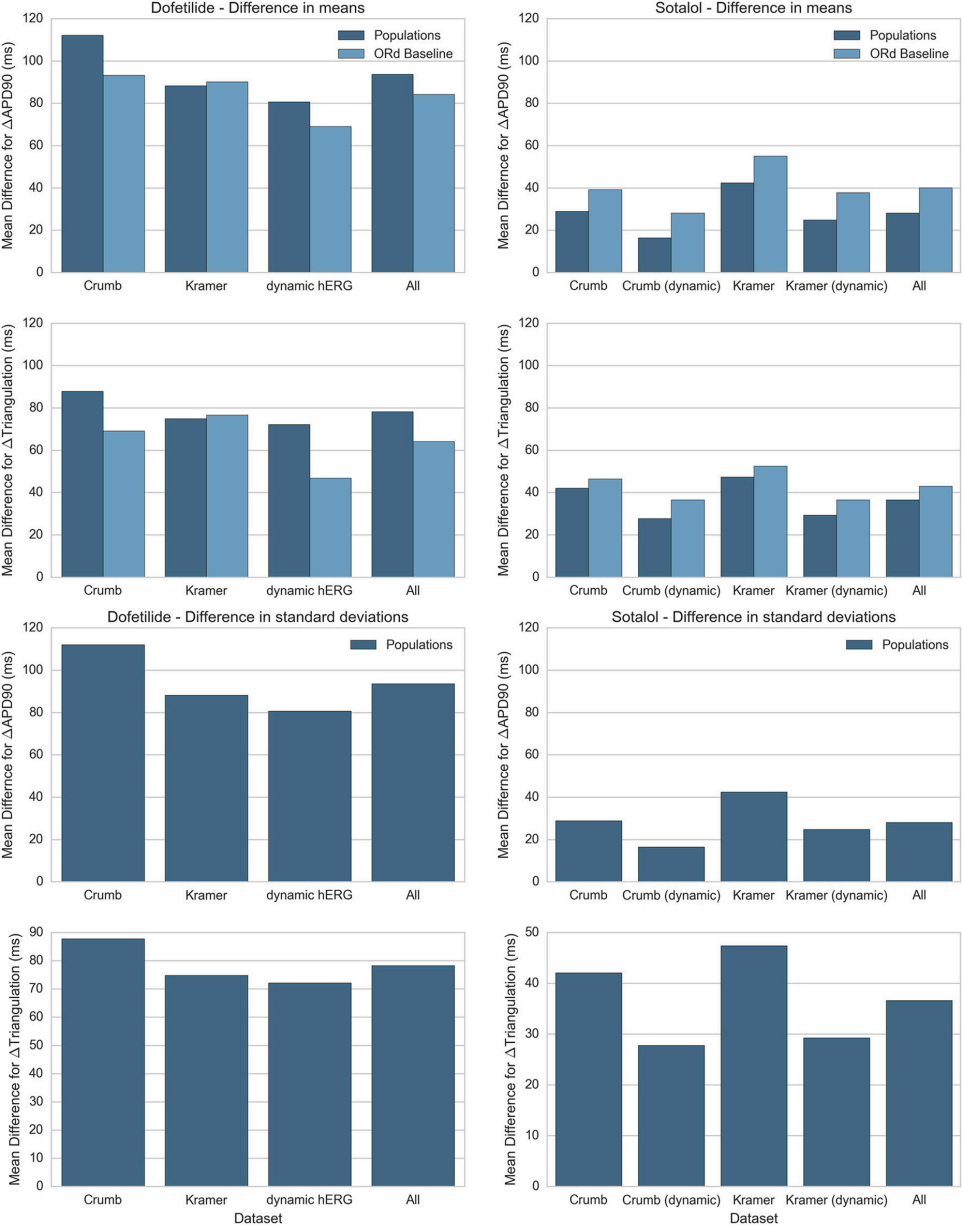
Summary of average difference in mean and standard deviation between experiment and simulation for ΔAPD90 and ΔTriangulation. Absolute differences in mean **(top)** and standard deviation **(bottom)** between experiments and simulations for ΔAPD90 and ΔTriangulation are shown for dofetilide **(left)** and sotalol **(right)**, for each drug block dataset. Differences in mean are shown for both the mean of the populations of models (dark blue) and the single biomarker value produced by the ORd baseline model (light blue), while differences in standard deviation can only be shown for the populations of models. Values for each drug block dataset are averaged across all concentrations and frequencies used in this study. Results for dofetilide show only one block dataset for the dynamic hERG model as neither Crumb nor Kramer IC50 datasets contained non-hERG IC50s for dofetilide (for sotalol, Kramer et al. measured an IC50 for Cav 1.2).

In this study we simulated two different IC50 datasets and two different models of I_Kr_ and I_Kr_ block, each combination of which produced a different simulated response to each drug. In addition we performed simulations with both populations of experimentally-calibrated models, and the baseline ORd model. Figure 9 summarizes the differences between simulation predictions and experimental results for the mean and standard deviation of ΔAPD90 and ΔTriangulation, separated by drug, drug block dataset, and modeling methodology. Figure 9 shows results for dofetilide and sotalol only as for quinidine and verapamil, all drug block datasets have the same qualitative mismatch with experiments. This makes a quantitative comparison redundant—all simulations can be thought of as being equally mismatched for these drugs.

We see from this comparison that the overall best drug block dataset for predicting ΔAPD90 and ΔTriangulation tested in this study is the dynamic I_Kr_ and I_Kr_ block model by Li et al. using the IC50s from Crumb et al. for non-hERG channels. In particular, the dynamic I_Kr_ model is consistently better than the default ORd I_Kr_ model using both Crumb and Kramer IC50s in all tested cases for both dofetilide and sotalol. Comparisons between the ORd baseline model and average of the populations of models are inconclusive: the baseline ORd model is closer to experiments for dofetilide, while the average of the populations of models is closer for sotalol. However, only the populations of models and not the baseline ORd model can provide predictions on variability of drug response.

## Discussion

### Main findings

In this study, changes in repolarization biomarkers and EAD occurrence caused by application of dofetilide, sotalol, quinidine, and verapamil were compared between *in silico* simulations using populations of human ventricular models and *ex vivo* human ventricular trabeculae. The four reference drugs examined in this study all blocked hERG but included both selective and multichannel blockers, as well as drugs in high and low TdP risk categories. Experimental data therefore spanned a wide range of effects from high APD prolongation with widespread EAD occurrence (dofetilide) to mild APD shortening with no EADs (verapamil). *In silico* populations of models were calibrated to reflect experimentally-observed AP variability between trabeculae from the same donor heart in control conditions, through variation in underlying ionic conductances. These populations were used to simulate the effects of each drug at multiple pacing rates and concentrations using IC50 data from two recent studies (Kramer et al., 2013; Crumb et al., 2016), and with both the ORd model's original model of hERG, and a recently developed state-dependent dynamic model of hERG and hERG block (Li et al., 2017) that integrates additional drug-specific data on hERG binding rates and trapping to model state-dependent block.

The main findings of this study were:

1. Comparison of *in silico* and *ex vivo* results showed overall agreement in APD90 and triangulation increase and EAD occurrence for both selective hERG blockers (dofetilide and sotalol). These drugs caused high APD prolongation, and dofetilide also caused EAD occurrence at the higher tested concentration (0.1 μM), while sotalol did not. These behaviors were all replicated at both pacing frequencies for sotalol and at 1 Hz for dofetilide. At 2 Hz EADs were not seen in dofetilide simulations, and simulated APD prolongation was lower overall than experiments due to skipping behavior, resulting in APs that were longer than one pacing cycle, which did not occur in simulations.
2. Experimental results for quinidine and verapamil, both multichannel blocking drugs, were generally not in agreement with simulations, due to prediction by simulations of substantially higher AP prolongation than observed experimentally. Although both quinidine and verapamil have untrapped hERG binding dynamics (Zhang et al., 1999; Tsujimae et al., 2004; Li et al., 2017), simulations using the recent state- and voltage-dependent model of I_Kr_ by Li et al. (2017) did not substantially improve agreement with experiments or reduce AP prolongation. However verapamil simulations did not develop any repolarization abnormalities such as EADs, in agreement with experiments. Quinidine simulations showed a very high incidence of repolarization abnormalities using the Crumb IC50s but a substantially lower incidence with the Kramer IC50s, most probably because the Crumb et al. study calculated IC50s for multiple potassium currents but was not able to reach a measurable IC50 for either Nav 1.5 (I_NaF_) or Cav 1.2 (I_CaL_), while the Kramer et al. study calculated IC50s for Nav 1.5 and Cav 1.2 as well as hERG. Overall, results for quinidine and verapamil suggest that inclusion of channel binding dynamics in the hERG block model are not sufficient to bring *in silico* results in line with experiments at the concentrations and frequencies tested, and suggest further studies are necessary to understand the biophysical mechanisms of these drugs' electrophysiological effects.

### Explanations for qualitative mismatch of APD changes but consistency in lack of EADs caused by verapamil

*Ex vivo* (Figure 8) and *in vivo* recordings (Johannesen et al., 2014; Vicente et al., 2015) show that verapamil causes minor QT and APD shortening or no effect. However, voltage clamp studies have consistently reported substantial hERG block in the concentration range tested in this study (Kramer et al., 2013; Crumb et al., 2016; Li et al., 2017). There are two main hypotheses in the literature regarding the lack of APD prolongation from verapamil despite this measured hERG block. The first hypothesis is that block of I_CaL_ by verapamil counteracts the AP prolonging effects of I_Kr_ block as both inward and outward currents are reduced, which produces the observed minor shortening of APD. However, the effects of I_Kr_ block alone are substantial and variable (e.g., Figures 5, 6). Therefore, it seems that this mechanism would require fine tuning of the ratios of I_CaL_ and I_Kr_ block, as well as the baseline cellular conductances G_Kr_ and G_CaL_, to consistently allow I_CaL_ block to cancel out the effects of I_Kr_ block alone, which multiple voltage clamp studies predict to be substantial at the concentrations tested. For example, for 1 μM verapamil both Crumb and Kramer datasets predict greater I_Kr_ block (68 and 77% respectively) than for 100 μM sotalol, the effects of which can be seen in Figure 6. It therefore seems unlikely that block of I_CaL_ could be a sufficient mechanism to precisely cancel out the AP prolongation from hERG block. However, verapamil's I_CaL_ block could be one of several contributing factors that collectively limit the AP prolongation from its I_Kr_ block, and experimental and *in silico* studies indicate it is the main mechanism preventing the occurrence of EADs under verapamil application.

The second hypothesis for verapamil's effects on APD is that I_Kr_ block from verapamil is overestimated by dose-response curve models parameterized from voltage clamp experiments that do not measure its hERG binding dynamics. Data from Zhang et al. (1999) show that verapamil is an untrapped hERG blocker–when bound it reduces the probability of the hERG channel closing, increasing the probability of verapamil unbinding at voltages close to the resting membrane potential. This contrasts with other hERG blockers, such as dofetilide, that do not prevent the channel from closing and therefore remain bound when the membrane is polarized. Therefore, IC50s measured from voltage clamp studies that do not account for this may overestimate the level of I_Kr_ block, and therefore the level of AP prolongation, caused by verapamil under normal pacing conditions. A simulation study by Di Veroli et al. (2014) suggests that verapamil's increased unbinding from hERG relative to compounds such as dofetilide could result in reduced AP prolongation during normal pacing, depending on binding timescales. However, use of the dynamic hERG model incorporating verapamil's untrapped dynamics did not substantially lower AP prolongation. Therefore, the mechanism(s) that limit the impact of verapamil's measured I_Kr_ block are currently unclear.

### Mismatch in APD prolongation and EAD occurrence for quinidine

Experimentally, quinidine causes QT and AP prolongation (Nademanee et al., 1990; Vicente et al., 2015), and is classified as a high risk torsadogenic drug. These features were qualitatively replicated in simulations (Figure 7); however the degree of AP prolongation was overestimated by simulations compared to *ex vivo* results. Additionally, while no EADs were recorded from any trabeculae under quinidine application, repolarization abnormalities occurred for the majority of models when using the IC50s from Crumb et al. in which only potassium channel IC50s were able to be calculated for quinidine, as recorded blocks of I_Na_ and I_CaL_ at the maximally tested concentration were too low to estimate IC50s, and in a small minority of models when using the IC50s from Kramer et al. which included block of I_CaL_ which is known to suppress EAD formation.

We can suggest four possible hypotheses for why simulations overestimated quinidine-induced AP prolongation. Firstly, as quinidine significantly blocks a particularly large number of channels, the compound effects of measurement uncertainty across multiple channels could result in a substantial total uncertainty when all channel blocks are integrated into an action potential model. Estimates of the hERG IC50 for the same drug across different studies have been shown to vary by an order of magnitude (Polak et al., 2009), and for a multichannel blocker like quinidine, this measurement uncertainty will be compounded over multiple ion channels. The second hypothesis is that the I_Kr_ current in the ORd model could have too large an influence on APD relative to other currents. However, the results for dofetilide and sotalol (Figures 5, 6) show that over a range of different conductance profiles the ORd model provides good agreement with experiments for selective block of I_Kr_ across multiple compounds, which provides confidence that the strength of I_Kr_ relative to other currents is reasonable. Thirdly, inward currents acting during repolarization, particularly I_CaL_, may be too weak in the ORd model, so that block of these currents produces too little reduction in APD prolongation when combined with I_Kr_ block. This could be tested in future studies by comparing simulations to experiments with more selective calcium channel blockers. If APD shortening in experiments is found to be substantially larger than in simulations using the ORd model, this would support this hypothesis. Finally, quinidine is known to be an untrapped hERG blocker (Tsujimae et al., 2004), so simple-pore block models may overestimate the degree of hERG block. However, quinidine binds rapidly to hERG channels (Windley et al., 2017), which would limit the effects of transient unbinding, and simulations with the dynamic hERG model did not show substantial differences to using only IC50 data (Figure 7). Therefore, the causes of mismatch between experiment and simulations for quinidine in our study require further investigation and could include a range of contributing factors.

### Limitations

This study investigated the response of models derived from a single baseline model, the ORd model, although with two different models of I_Kr_ and a wide range of different conductance profiles, to mimic biological variability in ion channel densities. Other sources of variability that are known to influence the electrophysiological phenotype, such as alterations in channel structure to change gating dynamics, are not included in this study. Other human ventricular models (e.g., ten Tusscher and Panfilov, 2006; Grandi et al., 2010) also have different balances of ionic currents and therefore produce different results in simulations and have different strengths and weaknesses. In particular, we found that across a wide range of conductances the ORd model could not reproduce the range of action potential amplitudes observed in this dataset, which were in the range of 87–119 mV (Figure 2). It is possible that the discrepancy in action potential amplitude could impact repolarization and ideally a modification to the model could be found to rectify this issue, but we have not yet found an appropriate modification. However, the ORd model was chosen as the baseline model for this study due to its integration of human-specific voltage-clamp and current-clamp recordings from human ventricular cardiomyocytes, and its current relevance for *in silico* drug testing due to being selected as the model of choice for the *in silico* section of CiPA (Fermini et al., 2016).

To incorporate inter- and intra-heart variability into simulation predictions, we chose to use the population of models methodology. However, other methodologies for integrating biological variability into cardiac modeling have been developed and could have been used, including multivariate partial regression analysis (Sobie, 2009; Sarkar and Sobie, 2010; Sadrieh et al., 2013) and particularly cell-specific modeling (Davies et al., 2012; Groenendaal et al., 2015). Each of these methodologies has particular strengths, e.g., partial least squares regression analysis can constrain model parameters and identify relationships between many model parameters and outputs simultaneously without the need for additional experimental data while cell-specific modeling can estimate best-fit parameter sets for recordings from specific cells, and can take advantage of information from dynamically rich pacing protocols (Groenendaal et al., 2015). The advantage of using cell-specific modeling in this study would have been the ability to find a unique model that agreed with the experimental recordings for each trabecula. However, the likelihood of each model accurately representing conductances of the associated preparation would be low as experimental recordings typically recorded from human preparations, such as those available here, would not contain enough information to constrain each model. These techniques are still under investigation.

Instead, we decided populations of models were a good choice of methodology for the purposes of this study. Although populations of models do not reconstruct the conductances of a particular preparation, they can find models with a wide range of ionic profiles that are all consistent with experimental biomarkers. This is ideal for simulating drug effects, as a wide range of possible responses, including outliers, can be evaluated. If the response to a simulated drug is different to experimental results across all or most models, as with quinidine and verapamil, this can then suggest that the mismatch is due to other causes, such as the model of drug block, or non-conductance sources of variability, rather than the specific set(s) of conductances in one or a few models. In addition, all current methods for incorporating experimental variability rely on the equations of an underlying baseline cell model such as the ORd model. Regardless of which model is chosen, there will likely be experimentally observed combination of AP biomarkers across different pacing protocols that cannot be simultaneously reproduced by a model with any set of conductances, due to the structure of the model equations. Therefore, no matter which methodology is used it may not be possible for all experimental observations to be reproduced in simulations with a single set of underlying model equations. Our study yields important quantitative information on the ability of the ORd model with variations in ionic conductance and current knowledge on drug action to reproduce experimental recordings.

The simple pore block model of drug action assumes that channel block is independent of the state of each ion channel. For many drugs this is an effective approximation; however for others, incorporating state-dependent block and binding information could be necessary to explain mismatches between simulations and experiments. Therefore, we also evaluated the state-dependent hERG block model by Li et al. (2017). Use of this model did not result in substantial changes in simulation results compared to the ORd baseline hERG model, however only one parameterization of this block model was available for each drug. Given the substantial uncertainty in measurements of IC50 values it is likely there is also substantial uncertainty in the drug block parameters measured for the Li et al. model. Replications of the type of voltage clamp studies used to parameterize these drug block models would be necessary to determine the level of uncertainty in hERG binding and trapping parameters, combined with further simulation studies to understand the effects this uncertainty has when propagated to AP-level models.

### Future work

The identification of mismatches between experiments and simulations is vital for continued improvement of *in silico* cardiac models and for identifying areas where our understanding of electrophysiological mechanisms of drug action is inconsistent with experimental data. We hope this study will motivate combined experimental and simulation studies that can explain the causes of the mismatches for quinidine and verapamil, and in doing so allow iterative modification and improvement of the ORd model and other cardiac cell models. This iterative improvement has been an important part of cardiac electrophysiology from the beginning of the field (Noble, 2011).

Future studies could also build on this work by analyzing a wider range of drugs, particularly other multichannel blockers, and selective blockers of channels other than hERG. This would provide a more thorough understanding of agreement and disagreement across a broad range of ion channel blocking compounds, providing confidence where selective block showed good agreement between simulations and experiments (e.g., as for dofetilide and sotalol in this study) and identifying areas for model modification where there is significant mismatch. In particular, it will be important to investigate whether the results for quinidine and verapamil are representative of other multichannel blockers that block hERG, or are outliers due to unique features of these two drugs. If the former, then it is likely the ORd model will need modification, if the latter, then the mismatch may be due to an incomplete understanding of the mechanisms of verapamil and quinidine block.

## Ethics statement

All human tissues used for this study were obtained by legal consent from organ donors in the United States.

## Author contributions

Conceived and designed study: OB, NA, BR. Acquired experimental recordings: NA, GP, AG, PM. Performed simulations and analyzed data: OB. Drafted manuscript: OB, NA, BR. All authors contributed to critical revision of the manuscript and approved the final version to be published.

### Conflict of interest statement

OB and BR authors declare no conflicts of interest. NA, GP, AG and PM are employees of AnaBios Corporation.
