# Supplementary material for: Quantitative Comparison of Effects of Dofetilide, Sotalol, Quinidine, and Verapamil between Human Ex vivo Trabeculae and In silico Ventricular Models Incorporating Inter-Individual Action Potential Variability
Source: Front Physiol. 2017 Aug 18;8:597. doi: 10.3389/fphys.2017.00597 (PMC5563361; doi:10.3389/fphys.2017.00597)
Supplement: Supplementary file 1 [file DataSheet1.docx]

**Supplementary Material**

**Supplementary Figures**

**Figure S1:** Example action potential traces from human left ventricular trabeculae recordings. Control recordings at 1 and 2 Hz are shown from five representative trabeculae from different donor hearts. The traces shown were from the trabeculae with the minimum, maximum and 25/50/75^th^ percentile values of APD90 across all trabeculae in the dataset.


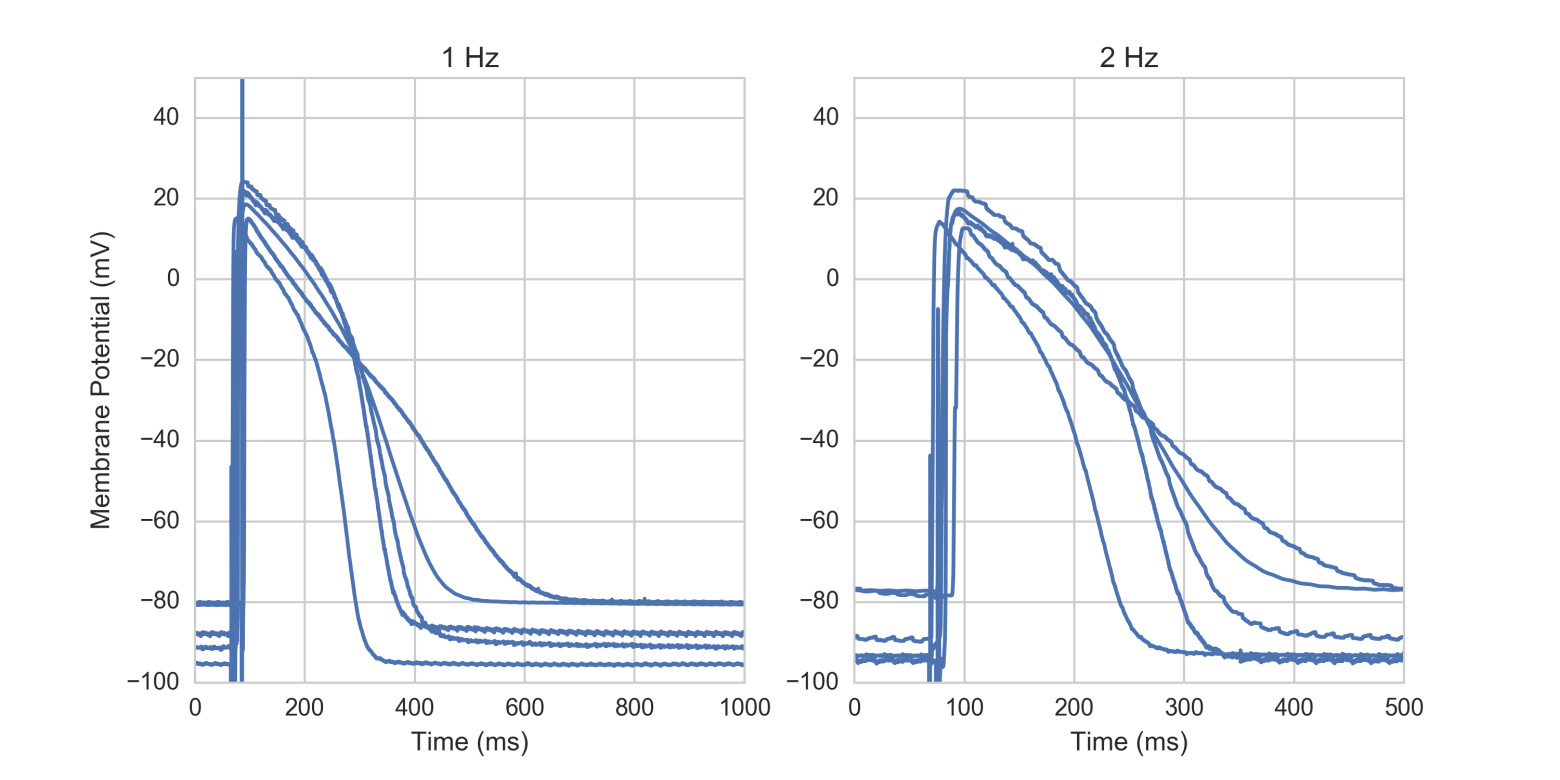


**Supplementary Tables**

**Table S1:** Drug blocks calculated for each drug and simulation, from Crumb et al. (2016), and Kramer et al. (2013). Blocks are given as fractions of remaining current after block (e.g. a value of 0.1 means the current was 90% blocked). Use of the dynamic hERG block model from Li et al. produced state-, time- and voltage-dependent block of IKr, and so hERG block varied between simulations and over time within each simulation.

| Drug | Concentration | Crumb | Kramer |
| --- | --- | --- | --- |
| Dofetilide | **0.01 μM** | **IKr: 0.201** | **IKr: 0.789** |
|  | **0.1 μM** | **IKr: 0.0594** | **IKr: 0.191** |
| Sotalol | **10 μM** | **IKr: 0.874** | **IKr: 0.853, ICaL: 0.951, INaF: 0.999** |
|  | **100 μM** | **IKr: 0.467** | **IKr: 0.52, ICaL: 0.659, INaF: 0.986** |
| Quinidine | **1 μM** | **IKr: 0.255, Ito: 0.835, IKs: 0.902** | **IKr: 0.414, ICaL: 0.779, INaF: 0.963** |
|  | **10 μM** | **IKr: 0.0332, Ito: 0.203, IKs: 0.269** | **IKr: 0.0579, ICaL: 0.425, INaF: 0.613** |
| Verapamil | **0.1 μM** | **IKr: 0.854, ICaL: 0.684** | **IKr: 0.693 , ICaL: 0.474** |
|  | **1 μM** | **IKr: 0.318, ICaL: 0.147** | **IKr: 0.226, ICaL: 0.125** |

**Table S2:** Changes to all models from all populations (n=860) from adoption of dynamic hERG model in control conditions. Biomarker values are given as the root mean squared values of the differences between biomarkers calculated from control simulations at 1 Hz pacing with models using the ORd model’s original I_Kr_ model, and with the dynamic I_Kr_ model by Li et al. (2017).

|  | APD10 (ms) | APD30 (ms) | APD50 (ms) | APD70 (ms) | APD90 (ms) | Triangulation (ms) (APD90-APD30) | RMP (mV) | APA (mV) | Max Neg  Slope (mV/s) |
| --- | --- | --- | --- | --- | --- | --- | --- | --- | --- |
| mean | 2.92 | 3.24 | 1.87 | 2.36 | 2.59 | 2.53 | 0.0214 | 0.255 | 12.1 |
| standard  deviation | 1.86 | 3.11 | 4.23 | 5.10 | 5.49 | 1.88 | 0.0213 | 0.103 | 12.8 |
| min | 0.001 | 0.26 | 0.0 | 0.0 | 0.0 | 0.0 | 0.0001 | 2.84 | 0.0 |
| 25% | 1.83 | 2.04 | 0.188 | 0.33 | 0.37 | 1.55 | 0.007 | 0.185 | 6.0 |
| 50% | 2.783 | 2.44 | 0.45 | 0.6 | 0.745 | 2.384 | 0.013 | 0.257 | 10.0 |
| 75% | 3.73 | 3.02 | 1.21 | 1.59 | 1.79 | 3.09 | 0.028 | 0.331 | 14.0 |
| max | 21.9 | 43.3 | 54.9 | 64.9 | 70.2 | 26.9 | 0.14 | 0.549 | 173.0 |

**Table S3:** Drug-induced changes to APD90. Change in APD90 for each drug and concentration tested (change relative to control), for 1 and 2 Hz pacing. Results are given as mean and standard deviation. Results for dofetilide are identical for the two dynamic I_Kr_ results as dofetilide only had a measured block of I_Kr_ for both datasets.

| Drug | Pacing rate (Hz) | Concentration (μM) | ΔAPD90,  Trabeculae  (ms) | ΔAPD90,  Crumb,  ORd I_Kr_ (ms) | ΔAPD90,  Crumb,  dynamic I_Kr_ (ms) | ΔAPD90,  Kramer,  ORd I_Kr_ (ms) | ΔAPD90,  Kramer,  dynamic I_Kr_ (ms) |
| --- | --- | --- | --- | --- | --- | --- | --- |
| Dofetilide | **1 Hz** | **0.01** | 104 (25) | 276 (56) | 185 (50) | 43 (5) | 185 (50) |
|  |  | **0.1** | 290 (70) | 346 (147) | 358 (169) | 283 (58) | 358 (169) |
|  | **2 Hz** | **0.01** | 108 (64) | 151 (21) | 117 (35) | 17 (3) | 117 (35) |
|  |  | **0.1** | 343 (92) | 164 (21) | 177 (32) | 149 (38) | 177 (32) |
| Sotalol | **1 Hz** | **10** | 48 (40) | 23 (2) | 34 (4) | 25 (3) | 32 (3) |
|  |  | **100** | 155 (42) | 133 (16) | 151 (18) | 101 (13) | 136 (17) |
|  | **2 Hz** | **10** | 43 (48) | 16 (2) | 25 (3) | 17 (2) | 23 (2) |
|  |  | **100** | 133 (74) | 91 (11) | 102 (12) | 66 (9) | 88 (11) |
| Quinidine | **1 Hz** | **1** | 2 (19) | 244 (43) | 171 (24) | 144 (22) | 156 (24) |
|  |  | **10** | 42 (23) | 70 (263) | 206 (277) | 379 (98) | 360 (81) |
|  | **2 Hz** | **1** | 1 (16) | 152 (25) | 110 (15) | 94 (15) | 98 (15) |
|  |  | **10** | 33 (22) | 107 (95) | 137 (89) | 174 (58) | 167 (64) |
| Verapamil | **1 Hz** | **0.1** | -16 (20) | 14 (7) | 19 (8) | 40 (13) | 5 (11) |
|  |  | **1** | -15 (30) | 148 (32) | 198 (43) | 195 (40) | 194 (43) |
|  | **2 Hz** | **0.1** | -9 (22) | 6 (5) | 16 (5) | 21 (10) | 4 (7) |
|  |  | **1** | -7 (23) | 82 (36) | 115 (47) | 103 (50) | 112 (46) |

**Table S4:** Drug-induced changes to Triangulation (APD90 – APD30). Change in Triangulation for each drug and concentration tested (change relative to control), for 1 and 2 Hz pacing. Results are given as mean and standard deviation. Results for dofetilide are identical for the two dynamic I_Kr_ results as dofetilide only had a measured block of I_Kr_ for both datasets.

| Drug | Pacing rate (Hz) | Concentration (μM) | ΔTri,  Trabeculae  (ms) | ΔTri,  Crumb,  ORd I_Kr_ (ms) | ΔTri ,  Crumb,  dynamic I_Kr_ (ms) | ΔTri ,  Kramer,  ORd I_Kr_ (ms) | ΔTri ,  Kramer,  dynamic I_Kr_ (ms) |
| --- | --- | --- | --- | --- | --- | --- | --- |
| Dofetilide | **1 Hz** | **0.01** | 77 (26) | 211 (45) | 143 (38) | 29 (4) | 143 (38) |
|  |  | **0.1** | 250 (78) | 289 (97) | 309 (119) | 218 (48) | 309 (119) |
|  | **2 Hz** | **0.01** | 80 (32) | 105 (32) | 98 (26) | 13 (2) | 98 (26) |
|  |  | **0.1** | 258 (80) | 106 (33) | 113 (46) | 104 (39) | 113 (46) |
| Sotalol | **1 Hz** | **10** | 41 (33) | 12 (2) | 22 (3) | 15 (2) | 22 (3) |
|  |  | **100** | 139 (42) | 81 (12) | 105 (14) | 65 (8) | 102 (12) |
|  | **2 Hz** | **10** | 33 (33) | 10 (1) | 17 (2) | 11 (1) | 17 (2) |
|  |  | **100** | 117 (53) | 59 (8) | 74 (9) | 49 (5) | 72 (8) |
| Quinidine | **1 Hz** | **1** | 11 (23) | 165 (33) | 121 (19) | 91 (14) | 112 (18) |
|  |  | **10** | 48 (18) | 135 (170) | 207 (173) | 245 (69) | 233 (60) |
|  | **2 Hz** | **1** | 5 (17) | 103 (17) | 80 (11) | 65 (10) | 75 (11) |
|  |  | **10** | 35 (15) | 108 (47) | 115 (50) | 105 (42) | 105 (44) |
| Verapamil | **1 Hz** | **0.1** | -3 (11) | 14 (4) | 18 (4) | 35 (7) | 16 (6) |
|  |  | **1** | 4 (23) | 121 (21) | 168 (33) | 156 (28) | 169 (33) |
|  | **2 Hz** | **0.1** | 2 (12) | 10 (3) | 17 (3) | 26 (5) | 15 (4) |
|  |  | **1** | 8 (20) | 86 (23) | 111 (37) | 104 (36) | 111 (36) |

**Table S5:** Drug-induced occurrence of repolarisation abnormalities (RAs). Number of trabeculae/models that developed EADs and other forms of repolarisation abnormality for each drug and concentration tested, for 1 and 2 Hz pacing. Results for dofetilide are identical for the two dynamic I_Kr_ results as dofetilide only had a measured block of I_Kr_ for both datasets.

| Drug | Pacing rate (Hz) | Concentration (μM) | RAs,  Trabeculae | RAs Crumb,  ORd I_Kr_ | RAs,  Crumb,  dynamic I_Kr_ | RAs,  Kramer,  ORd I_Kr_ | RAs,  Kramer,  dynamic |
| --- | --- | --- | --- | --- | --- | --- | --- |
| Dofetilide | **1 Hz** | **0.01** | 0/9 | 0/26 | 0/26 | 0/26 | 0/26 |
|  |  | **0.1** | **5/9** | **4/26** | **4/26** | 0/26 | 0/26 |
|  | **2 Hz** | **0.01** | 0/9 | 0/26 | 0/26 | 0/26 | 0/26 |
|  |  | **0.1** | **5/9** | **4/26** | **4/26** | 0/26 | 0/26 |
| Sotalol | **1 Hz** | **10** | 0/15 | 0/459 | 0/459 | 0/459 | 0/459 |
|  |  | **100** | 0/15 | 0/459 | 0/459 | 0/459 | 0/459 |
|  | **2 Hz** | **10** | 0/15 | 0/459 | 0/459 | 0/459 | 0/459 |
|  |  | **100** | 0/15 | 0/459 | 0/459 | 0/459 | 0/459 |
| Quinidine | **1 Hz** | **1** | 0/15 | **1/501** | 0/501 | 0/501 | 0/501 |
|  |  | **10** | 0/15 | **421/501** | **334/501** | **15/501** | **8/501** |
|  | **2 Hz** | **1** | 0/15 | 0/501 | 0/501 | 0/501 | 0/501 |
|  |  | **10** | 0/15 | **151/501** | **75/501** | 0/501 | 0/501 |
| Verapamil | **1 Hz** | **0.1** | 0/14 | 0/689 | 0/689 | 0/689 | 0/689 |
|  |  | **1** | 0/14 | 0/689 | 0/689 | 0/689 | 0/689 |
|  | **2 Hz** | **0.1** | 0/14 | 0/689 | 0/689 | 0/689 | 0/689 |
|  |  | **1** | 0/14 | 0/689 | 0/689 | 0/689 | 0/689 |
